# Supplementary material for: MetaQTL: a package of new computational methods for the meta-analysis of QTL mapping experiments
Source: BMC Bioinformatics. 2007 Feb 8;8:49. doi: 10.1186/1471-2105-8-49 (PMC1808479; doi:10.1186/1471-2105-8-49)
Supplement: Additional File 7 — MetaQTL Package : jar file and tutorial. This Zip archive contains both the MetaQTL JAR file and the files of the tutorial. [file 1471-2105-8-49-S7.zip › org.inra.metaqtl/doc/org/metaqtl/ChromCluster.ClusterEdge.html]

ChromCluster.ClusterEdge


|  |  |  |
| --- | --- | --- |
| **Overview** \| **Package** \| **Class** \| **Use** \| **Tree** \| **Deprecated** \| **Index** \| **Help** | |  |
| **PREV CLASS**   **NEXT CLASS** | **FRAMES**    **NO FRAMES**     **All Classes** |  |
| SUMMARY: NESTED \| FIELD \| CONSTR \| METHOD | DETAIL: FIELD \| CONSTR \| METHOD |  |


---


## org.metaqtl Class ChromCluster.ClusterEdge

```
java.lang.Object
  org.metaqtl.ChromCluster.ClusterEdge
```

**Enclosing class:**: ChromCluster

---

``` public class ChromCluster.ClusterEdge extends java.lang.Object ```

---

| **Field Summary** | |
| --- | --- |
| `Chromosome` | `chrom` |
| `CMarkerSequence` | `cms` |


**Constructor Summary**

`ChromCluster.ClusterEdge(CMarkerSequence cms,
Chromosome chrom)`


**Method Summary**

**Methods inherited from class java.lang.Object**

`clone, equals, finalize, getClass, hashCode, notify, notifyAll, toString, wait, wait, wait`

**Field Detail**

### cms

```
public CMarkerSequence cms
```

---


### chrom

```
public Chromosome chrom
```


**Constructor Detail**

### ChromCluster.ClusterEdge

```
public ChromCluster.ClusterEdge(CMarkerSequence cms,
                                Chromosome chrom)
```

**Parameters:**: `nmc` -: `cur2` -


---


|  |  |  |
| --- | --- | --- |
| **Overview** \| **Package** \| **Class** \| **Use** \| **Tree** \| **Deprecated** \| **Index** \| **Help** | |  |
| **PREV CLASS**   **NEXT CLASS** | **FRAMES**    **NO FRAMES**     **All Classes** |  |
| SUMMARY: NESTED \| FIELD \| CONSTR \| METHOD | DETAIL: FIELD \| CONSTR \| METHOD |  |


---
